# Self-poisoning in rural Sri Lanka: small-area variations in incidence

**DOI:** 10.1186/1471-2458-8-26

**Published:** 2008-01-23

**Authors:** Celie Manuel, David J Gunnell, Wim van der Hoek, Andrew Dawson, Ishika K Wijeratne, Flemming Konradsen

**Affiliations:** 1 Department of International Health, Institute of International health, Immunology and Microbiology, University of Copenhagen, Øster Farimagsgade 5, Building 16, P.O. Box 2099, 1014 Cph K, Denmark; 2 Department of Social Medicine, University of Bristol, Bristol, UK; 3 South Asian Clinical Toxicology Research Collaboration, University of Peradeniya, Sri Lanka; 4 Department of Clinical Medicine, University of Peradeniya, Sri Lanka; 5 School of Population Health, University of Newcastle, Australia; 6 Urban Development Authority, GIS Centre, "Sethsiripaya", Battaramulla, Sri Lanka

## Abstract

**Background:**

Self-poisoning is one of the most common methods of suicide worldwide. The intentional ingestion of pesticides is the main contributor to such deaths and in many parts of rural Asia pesticide self-poisoning is a major public health problem. To inform the development of preventive measures in these settings, this study investigates small-area variation in self-poisoning incidence and its association with area-based socioeconomic and agricultural factors.

**Methods:**

Ecological analysis of intentional self-poisoning in a rural area (population 267,613) of Sri Lanka in 2002. The geographic distribution of cases was mapped to place of residence. Using administrative division (GN), median population size 1416, as unit of analysis, associations with socioeconomic and agricultural indicators were explored using negative binomial regression models.

**Results:**

The overall incidence of intentional self-poisoning in the study area was 315 per 100,000 (range: 0 – 2168 per 100,000 across GNs). Socioeconomic disadvantage, as indexed by poor housing quality (p = 0.003) and low levels of education (p < 0.001) but not unemployment (p = 0.147), was associated with a low self-poisoning incidence. Areas where a high proportion of the population worked in agriculture had low overall levels of self-poisoning (p = 0.002), but a greater proportion of episodes in these areas involved pesticides (p = 0.01). An association with extent of cultivated land was found only for non-pesticide poisoning (p = 0.01).

**Conclusion:**

Considerable small-area variation in incidence rates of intentional self-poisoning was found. The noteworthy concentration of cases in certain areas and the inverse association with socioeconomic deprivation merit attention and should be investigated using individual-level exposure data.

## Background

Self-poisoning is one of the most common methods of suicide and self-harm worldwide. In many developing countries, the intentional ingestion of highly toxic yet easily available products, especially pesticides, causes a high burden of premature death and disability. An estimated 300,000 annual deaths from pesticide self-poisoning in Asia alone establishes this as a major public health problem [1].

Sri Lanka is a case in point. About 60% of suicides in Sri Lanka are caused by intentional self-poisoning and – of these – 90% are due to deliberate pesticide ingestion [2]. Most studies of self-poisoning in Sri Lanka are hospital based, focussing on patient characteristics or the toxicological aspects of ingested poisons [3-5]. Only a few have dealt with social determinants and cultural meanings of self-poisoning [6-8], while none to date have investigated whether area characteristics are associated with the incidence of self-poisoning and use of pesticides for self-harm. Little is known about the influence in Sri Lanka of social deprivation or availability of means, though these factors have been shown to be relevant determinants of self-harm and suicide elsewhere [9-11].

The clarification of such associations in rural Asia is essential to advance our understanding of the determinants of self-poisoning and thereby guide the development of appropriate preventive strategies.

This study investigates the geographical variations in incidence of self-poisoning, in particular pesticide self-poisoning, in a rural area of Sri Lanka. Furthermore, it explores the value of area-based agricultural and socioeconomic factors in explaining these variations.

## Methods

### Study area and data collection

**Figure 1:**
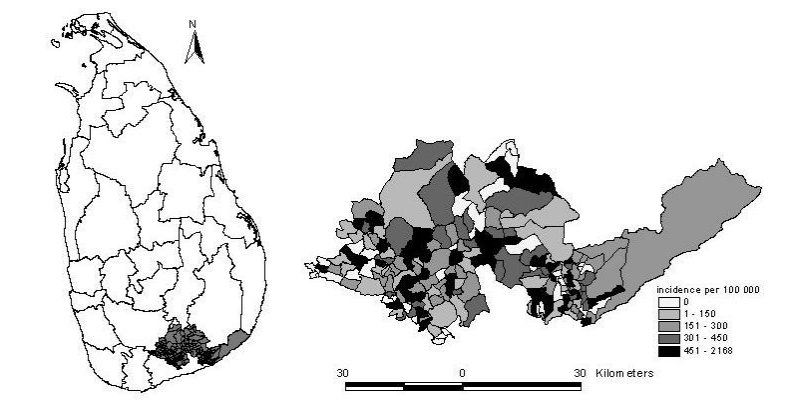
Intentional self-poisoning incidence per 100.000 inhabitants (2002).

The study area, with a population of 267,613 above ten years of age, is located in the dry-zone of southern Sri Lanka (see Figure 1). It is a rural agricultural area dominated by subsistence farmers involved in irrigated rice cultivation. Vegetables, bananas and sugarcane are also cultivated in some areas. In non-irrigated areas, traditional slash-and-burn cultivation is practiced in small plots of land ("chenas").

Data were collected for all self-poisoning admissions in 2002 to the nine government hospitals in the study area. The medical records of all in-patients were examined to identify intentional self-poisoning cases, as determined by the attending medical officer. Information was extracted on sex, age, place of residence, type of poison ingested, and outcome (discharged, transferred or died). Names of patients were not recorded due to ethical constraints, which makes data episode- rather than person-based. The same research assistant was responsible for the data collection from all hospitals.

Each case was assigned to a Grama Niladhari (GN) division (median population 1416, range 214 to 5804), the smallest administrative unit in Sri Lanka, according to the place of residence (i.e. village) recorded in their hospital records. For some cases, identification of the appropriate GN was complicated due to (i) ambiguous translations of Sinhala place names, and (ii) identically named villages located in different administrative areas. In such cases, maps of 1:50,000 scale from the Survey Department of Sri Lanka were examined to identify locations of villages and decide the specific GNs most likely (in terms of proximity) to present cases to the respective hospitals. For six villages, assignment to either of two neighbouring GNs was possible. Such GNs were combined to create single areas. Local government officials and hospital staff were asked to assist personally in the identification of GNs for the remaining unknown villages.

To ensure that all cases of hospital-presenting intentional self-poisoning from the geographic area were included in the study, the study area was restricted to those GNs where the majority of inhabitants were considered likely to visit only the hospitals covered by the data collection. Peripheral GNs were only included in the study area if the distance to the nearest hospital and the road infrastructure made it unlikely that inhabitants would seek treatment in out-of-area hospitals.

There were 929 admissions with intentional self-poisoning to the nine hospitals in 2002. Of these, 17 cases transferred between two hospitals *within *the study area and were therefore deleted from the file of the referring hospital to avoid double-counting. Cases residing in GNs not classified as belonging to the catchment area (n = 33), as well as cases for which a GN of residence could not be established (n = 35), were also excluded, reducing the number of self-poisoning cases in the study area to 844. In total there were 189 GNs in our main analysis.

### Exposure variables

To explore the association of intentional self-poisoning with area level characteristics, GN-based indicators of socioeconomic position were extracted from the 2001 national household census [12]. The variables included: (i) educational attainment (percentage of the population above 15 years having completed secondary school); (ii) unemployment (percentage of the economically active population who were unemployed); and (iii) a composite variable for housing quality incorporating census data on four indicators: permanence of outer wall material, availability of a water-seal toilet, protected source of drinking water and use of electricity as the principal source of lighting. Each variable in the composite index was dichotomised and given a score of either 0 or 1, with 1 indicating poorer housing quality. The final housing score was calculated as the sum of these four indicators.

In addition, two agricultural variables were included as crude markers of pesticide availability in line with other developing-country studies on suicide [10,13]. These were: (i) agricultural population – the percentage of the economically active population employed in agriculture and forestry as recorded in the 2001 national census; and (ii) the proportion of cultivated land. Land-use data are based on analysis of remotely sensed Landsat images from 2003 obtained from the International Water Management Institute (IWMI), Colombo, Sri Lanka.

### Analyses

Age group- and sex-specific incidence rates of intentional self-poisoning for each area were calculated using the census-derived population figures for each GN. The relatively small number of cases precluded examination of individual age-bands. Age was therefore recoded as '10–24 years' and '25 years and above', using the median age of cases as cut-off value. No cases of intentional self-poisoning below 10 years of age were reported.

We used STATA 8.0 (Stata Corp., College Station, TX) to investigate associations between area-based exposure variables and rates of intentional self-poisoning. To determine the most appropriate model for the data, we explored both Poisson and negative binomial regression models. Incidence rate ratios (IRRs) were estimated across quartiles of each exposure variable, using the lowest exposure as the reference category. Associations between self-poisoning and exposure were investigated for total self-poisoning, as well as for pesticide and non-pesticide self-poisoning separately, controlling for age and sex in all models.

To investigate the possible impact of cross-boundary treatment-seeking amongst people living in GNs on the boundary of the study area (i.e. that they might seek treatment in hospitals outside the study area and therefore not be identified in the data collection), a sensitivity analysis was conducted excluding all peripheral GNs (N = 45).

Maps of incidence were produced using the ArcView 3.3 Geographic Information System (GIS) software.

## Results

### Study sample

Altogether, 844 cases from the study catchment area presented to hospital for treatment of intentional self-poisoning during 2002. By far the most common poisons ingested (irrespective of outcome) were pesticides (55.8% of cases), the majority of which were organophosphorus insecticides. Other common ingestions were kerosene oil (10.2%), poisonous plants (8.5%) and medicinal drugs (7.6%). The type of poison was unidentifiable for 15.6% of cases and these were classified as non-pesticide self-poisonings in the analysis. Only one of the 844 cases had taken more than one type of poison simultaneously. Pesticides were the cause of 90% (37/41) of deaths.

### Incidence

The overall incidence of self-poisoning in the study area in 2002 was 315 per 100,000 inhabitants above ten years of age. Incidence in males was higher than in females (330 vs. 299 per 100,000) and more than twice as high in 10–24 year olds vs. those aged ≥25 (439 vs. 202 per 100,000). Intentional self-poisoning resulted in death in 41 cases, generating an overall fatal self-poisoning rate of 15.3 per 100,000.

The geographic distribution of self-poisoning in the study area, presented in Figure 1, shows some evidence of clustering. Over one fifth of GNs (40/189) had incidences of more than 500 per 100,000 inhabitants, thus accounting for 54% of cases (453/844) yet only 22% of the at-risk population. Nine GNs had incidence rates higher than 1000 per 100,000.

### Associations between self-poisoning incidence and exposure variables

Table 1 documents the socioeconomic and agricultural characteristics of the study area. The unemployment rate at the time of census was approximately 8%, and less than a quarter of those aged ≥15 years had completed secondary school education. Over half the population were involved in agricultural work.

**Table 1:** Distribution of area-based exposure variables

**Exposure**^1^					
	***N***	**Mean**	**SD**	**Median**	**Range**

Housing Quality (score out of 4)	*189*	1.68	1.36	1	(0–4)
Unemployment (%)	*189*	8.55	5.05	8	(0–27)
Education (% of >15 year olds completing 2^o ^school)	*189*	23.39	9.55	22	(6–54)
Agricultural Population (%)	*189*	57.20	21.2	63	(5–94)
Agricultural Land-use (%)	*185*^2^	71.01	31.47	87	(4–100)

^1^Exposure measured in 2001, except agricultural land-use measured in 2003

^2^Data on land-use were complete only for 185 GNs

To determine the best model for the data, we explored associations between intentional self-poisoning and the socioeconomic and agricultural exposure variables using both Poisson and negative binomial regression models. The differences in results for the two types of regression analyses were negligible. However, the analyses indicated statistically significant extra-Poisson variation (α > 0, p < 0.0001), and in the following, the results of the negative binomial regression analysis are therefore reported.

Table 2 shows the results of the regression analysis. There is clear evidence that the incidence of self-poisoning was lower in areas characterised by poorer socioeconomic conditions and where a large proportion of the population were employed in agriculture. Higher levels of unemployment and agricultural land-use were not associated with overall self-poisoning.

**Table 2:** Age- and sex-adjusted associations between self-poisoning and exposure variables

		**TOTAL 2002**			**PESTICIDES 2002**			**NON-PESTICIDES 2002**		
**Exposure**		IRR^1^	95CI	p-value^2^	IRR^1^	95CI	p-value^2^	IRR^1^	95CI	p-value^2^
**Housing Score**^3^	[0]	1			1			1		
	[1]	0.73	(0.56–0.96)		0.71	(0.52–0.97)		0.79	(0.56–1.12)	
	[2]	0.72	(0.53–0.98)		0.82	(0.58–1.16)		0.63	(0.42–0.95)	
	[3-4]	0.65	(0.51–0.84)	0.003	0.90	(0.68–1.20)	0.817	0.43	(0.30–0.62)	<0.001
**Education**^4^	(29–54%)	1			1			1		
	(22–28%)	0.83	(0.64–1.07)		0.78	(0.58–1.04)		0.90	(0.63–1.27)	
	(17–21%)	0.63	(0.48–0.82)		0.57	(0.42–0.77)		0.71	(0.50–1.02)	
	(6–16%)	0.61	(0.46–0.82)	<0.001	0.76	(0.55–1.05)	0.015	0.43	(0.27–0.67)	0.002
**Unemployment**	(0–4%)	1			1			1		
	(5–7%)	1.23	(0.92–1.64)		1.13	(0.83–1.54)		1.45	(0.96–2.19)	
	(8–10%)	1.23	(0.90–1.69)		1.07	(0.75–1.51)		1.52	(0.97–2.38)	
	(11–27%)	1.29	(0.96–1.72)	0.147	0.95	(0.68–1.33)	0.609	1.90	(1.26–2.86)	0.004
**Agricultural Population**	(5–44%)	1			1			1		
	(45–62%)	0.77	(0.59–0.99)		0.78	(0.58–1.05)		0.80	(0.57–1.12)	
	(63–73%)	0.72	(0.54–0.95)		0.88	(0.64–1.21)		0.56	(0.38–0.83)	
	(74–94%)	0.59	(0.45–0.78)	0.002	0.80	(0.59–1.08)	0.396	0.41	(0.28–0.61)	<0.001
**Agricultural land-use**	(0–44%)	1			1			1		
	(45–86%)	1.92	(1.44–2.56)		1.61	(1.17–2.20)		2.44	(1.59–3.74)	
	(87–98%)	1.17	(0.87–1.57)		0.83	(0.59–1.17)		1.78	(1.15–2.76)	
	(99–100%)	1.17	(0.88–1.56)	0.374	0.99	(0.72–1.36)	0.357	1.59	(1.03–2.44)	0.013

^1 ^All IRRs are adjusted for age and sex

^2 ^p-values are given for the association with the uncategorised variables. IRRs are given for each quartile of the exposure variable

^3 ^Housing score: a higher score denotes higher housing deprivation, i.e. poorer quality housing indicating lower socioeconomic status

^4 ^Education: percentage of the population above 15 yrs of age having completed secondary school, i.e. a lower percentage indicating lower socioeconomic status

Separate analyses for pesticide vs. non-pesticide self-poisoning, respectively, revealed that the overall patterns were mainly determined by associations with non-pesticide poisoning. Interestingly, all exposure variables, including unemployment and agricultural land-use, were strongly associated with non-pesticide poisoning, while only education maintained a significant association with pesticide self-poisoning (p = 0.015). Further analysis investigating the correlation between agricultural population and the proportion of self-poisonings that specifically involved pesticides showed, however, that pesticides were used in a greater proportion of self-poisoning episodes in areas where relatively larger proportions of the population were employed in agriculture (Spearman's rho = 0.17, p = 0.01).

In additional models, we examined whether the associations of self-poisoning with the agricultural factors might be confounded by socioeconomic conditions but found no indication of this. For example, the relative risk of self-poisoning in areas with larger agricultural populations adjusted for the effect of housing quality showed the same protective trend as pre-adjustment: 1.0, 0.78 (0.60–1.01); 0.75 (0.55–1.02); 0.63 (0.46–0.86), (p = 0.11). We further investigated whether associations differed in males and females, and between age groups, by adding interaction terms to the models. No evidence of effect modification was found; the p-value for interaction between sex and education with respect to their effect on overall rates of self-poisoning was p = 0.58; likewise, the p-value for interaction between age and education was p = 0.98.

A sensitivity analysis was conducted to determine whether exclusion of the 45 GNs around the border of the study area -and thereby the risk of cross-boundary treatment seeking – influenced our key findings. The overall self-poisoning rate in the peripheral GNs was 224 per 100,000, somewhat lower than that of the complete study area (315 per 100,000). Exclusion of these areas, however, did not materially alter any of the findings reported in Table 2. For example, the IRRs for the overall incidence of self-poisoning across categories of poorer housing quality were still higly significant: 1.00; 0.83 (0.62–1.10); 0.72 (0.52–1.00); 0.66 (0.49–0.88) p = 0.002 (other results not shown).

## Discussion

To the best of our knowledge, this is the first analysis of the geographic variations in the incidence of self-poisoning in rural Asia. Likewise, it is the first study in rural Asia to analyse the association of area-based socioeconomic and agricultural characteristics with the incidence of self-poisoning.

### Main findings

The overall rate of intentional self-poisoning in the study area was 315 per 100,000 inhabitants, confirming the high rates reported from other Sri Lankan studies [14,15]. The distribution of cases varied considerably across GNs (range 0–2168 per 100,000, median 234 per 100,000), and the disproportionately high contribution to self-poisoning incidence by a fairly small number of GNs suggests that the spatial variation may not be random. This is supported by the fact that nine of the top-incidence GNs in 2002 also ranked in the top 10% of self-poisoning incidence GNs in each of the preceding 3 years (1999–2001) [16]. However, as the numbers of self-poisoning cases per GN predominately remain quite small, no strong conclusions can be drawn.

Our investigations of the association of self-poisoning with area level markers of socioeconomic and agricultural conditions yielded surprising results. For instance, the finding that areas characterised by high levels of socioeconomic deprivation tended to have lower rates of self-poisoning is at odds with the research from high-income countries where area levels of socioeconomic deprivation are in general positively associated with risk of self-harm [9,17]. The role of socioeconomic deprivation in the aetiology of self-harm may well be different in rural Asia. Certainly, the causal pathway is complex, as suggested in this study by the divergence in associations of the different measures of socioeconomic status (education and housing vs. unemployment). Previous Sri Lankan studies support this view. For example, in a case control study of self-harm conducted in 1999, Thalagala and Fernando [18] reported an increased risk among those with higher levels of education (OR 1.8 95%CI 1.1 to 2.9), although low income was also associated with increased risk. Van der Hoek and Konradsen [19] in their case control study of pesticide self-poisoning conducted within the current study area reported no association between socioeconomic position or debt and self-poisoning, although they did find a protective effect of higher levels of education. The only previous ecological study of suicidal behaviour in Sri Lanka [20] examined associations with suicide at a high level of geographic aggregation (22 districts of Sri Lanka, total population 12,7 million). The author found no association between literacy or urbanization with suicide, but reported that high levels of unemployment were associated with lower suicide rates.

It is possible that different markers of socioeconomic deprivation are associated with self-harm through separate pathways and also that the effect of the various socioeconomic indicators may vary from context to context. For instance, in farming communities, unemployment may be of a seasonal nature affecting large parts of the community at the same time, thereby entailing fewer social and psychological consequences than in urbanised areas. Similarly, in theory, the composite indicator 'housing quality' may not expose the same aspects of socioeconomic status in urban and rural areas due to differences either in construction costs or community availability of electricity and protected drinking water.

Future studies of self-harm in developing countries should assess such issues and consider plausible explanatory pathways using individual level data.

The role of the agricultural factors is similarly complex. We found that GNs with a relatively high proportion of the population involved in agriculture had lower rates of self-poisoning, while the reverse association was seen in relation to the proportion of cultivated land.

This may be due to the variables not, in fact, being clear indicators of pesticide accessibility. 'Agricultural land-use' covers different types of crop with varying requirements of pesticide application, while 'population employed in agriculture' similarly includes farmers involved in different types of cultivation. Detailed information on specific crop-types from agricultural surveys as employed in a methodologically comparable study from Brazil [13] might have provided more accurate indicators of pesticide usage. Lin and Lu [10] have previously used 'percentage of the population employed in agriculture' as an indicator of pesticide accessibility in Taiwan. A strong positive association of this variable with suicide by intentional ingestion of pesticides, led the authors to conclude that the easy availability of pesticides influenced their usage for self-harm. In support of this, our findings show that the relative contribution of pesticide ingestion in self-poisoning episodes was larger in areas with large agricultural populations.

### Strengths and Limitations

We carried out detailed medical record searches to identify all cases of self-poisoning and used small-area level census and satellite image data to characterise the study area. The last census in Sri Lanka was carried out in 2001 and satellite images were taken in 2003, so our data on the characteristics of the study area correspond closely in time to the measurement of intentional self-poisoning. The low level of aggregation (median population 1416) more accurately describes the ecological exposure of the individuals studied, reducing though not entirely avoiding the inherent susceptibility of area-based studies using aggregate data to ecological bias. While using smaller areas for analysis gave a higher statistical power to detect associations (due to a larger number of analysed units), the small number of events per GN reduced precision of the incidence rate estimates. A further concern is the issue of spatial autocorrelation, if the patterns of self-poisoning in a GN are influenced by self-poisoning in neighbouring areas simply by virtue of their spatial proximity. Such correlation may potentially affect the results of the regression analyses, and it would be useful to assess and control for this in future studies [21].

There are a number of further limitations to our analysis. First, hospital admission data may not satisfactorily capture the incidence of self-poisoning in the community. Varying accuracy in hospital records may affect the identification of cases both with regard to whether self-poisoning was intentional or accidental, and with regard to the type of poison ingested. Also, hospital presentation may not occur in cases of minor poisoning or those episodes resulting in death before hospital treatment can be sought, while some severe poisonings may have been taken directly to larger better-equipped hospitals outside the study area. The associations with area characteristics are, however, only likely to be biased if missing data are more common in certain areas. For instance, if the lower rates of self-poisoning in areas of socioeconomic deprivation were observed simply due to reduced hospital access in such areas the resultant associations might entirely be the effect of selection bias. However, we find no reason to suppose that access to hospitals or treatment seeking might vary across the study area; hospital care is provided free of charge and all hospitals lie within a short and easy travelling distance of each other. Furthermore, our sensitivity analysis did not suggest an effect in peripheral GNs, where cross boundary treatment seeking might otherwise have been an issue. Second, we were unable to clearly identify repeated admissions for the same individual over the 12-month study period and this could potentially inflate rate estimates in some areas. Careful scrutiny of the case data with regard to sex, age and place of residence did not, however, suggest repetition of self-poisoning to be a problem. Third, some difficulties were encountered in assigning cases to GN-divisions. Any resulting misclassification is, however, likely to be random with respect to the exposure variables examined and so lead to an under-estimation of any associations.

## Conclusion

We found substantial variation in small-area incidence of intentional self-poisoning, with some areas contributing a disproportionately high and troubling number of cases. The resulting incidence rates reaching well over 1000 per 100,000 population are worthy of acute attention. The unexpected pattern of associations of area-level socioeconomic and agricultural factors with intentional self-poisoning makes interpretation of their influence difficult. Of note, there were no strong ecological associations with patterns of intentional pesticide poisoning – the class of poisons which, because of their high case-fatality and common use for self-harm, are the cause of most health concern in developing nations. Other, and more specific, determinants of self-harm behaviour in low- and middle income countries, which are presumably individual rather than contextual in nature, need to be explored. The identification of high-incidence areas could effectively be used to guide such future research efforts on determinants of intentional self-poisoning.

## Competing interests

The author(s) declare that they have no competing interests.

## Authors' contributions

CM, DG, WvdH, AHD and FK were involved in designing the study and the format for data collection. CM, WvdH and FK were responsible for the data collection. CM was mainly responsible for data handling and quality assurance. IKW facilitated access to secondary information, produced maps of the study area and provided inputs to the descriptive analysis. CM and DG conducted the data analysis. CM produced the first version of the draft paper and produced revisions following contributions from DG, WvdH, FK, and AHD. All authors approved the final manuscript.

## Pre-publication history

The pre-publication history for this paper can be accessed here:


